# Recurrent acute coronary syndrome in a patient with right coronary artery ectasia: a case report

**DOI:** 10.1186/s13256-019-1979-x

**Published:** 2019-03-09

**Authors:** Vito Damay, Raymond Pranata, Wendy Wiharja

**Affiliations:** 1 Faculty of Medicine, Universitas Pelita Harapan, Tangerang, Banten Indonesia; 2 Department of Cardiology and Vascular Medicine, Siloam Hospitals Lippo Village, Tangerang, Banten Indonesia

**Keywords:** Coronary artery ectasia, Acute coronary syndrome, Microvascular, Anticoagulant, Recurrence

## Abstract

**Background:**

Coronary artery ectasia is characterized by an abnormal dilatation of the coronary arteries. Coronary artery ectasia is observed in 3–8% of patients undergoing coronary angiography and sometimes leads to acute coronary syndrome regardless of the presence or absence of coronary stenosis or atrial fibrillation.

**Case presentation:**

A 61-year-old Indonesian man presented with typical angina that began 1 week before admission and had worsened 3 hours prior to admission. Accompanying symptoms included dyspnea, nausea, and sweating. He was hemodynamically stable and had a history of tobacco smoking and dyslipidemia. An electrocardiogram showed ST-segment depression and T inversion. Laboratory results showed an international normalized ratio of 1.28. Dual antiplatelet therapy was administered along with fondaparinux, and symptoms were alleviated. Coronary angiography showed an ectatic and turbulent mid-distal right coronary artery and slow flow at the first presentation. There was a patent stent in the proximal-mid left anterior descending coronary artery. This patient had previously presented with recurrent acute coronary syndrome and received two coronary stents for the stenotic vessels.

**Discussion:**

He had right coronary artery ectasia and experienced recurrent acute coronary syndrome. He received dual antiplatelet therapy along with warfarin after stenting of his left anterior descending coronary artery. However, he presented with unstable angina pectoris 7 months before the latest admission and at the latest admission despite a patent stent and no other significant obstructive lesion. The unstable angina pectoris might have been caused by slow flow, microvascular angina caused by small thrombi and/or vasospasm, or epicardial thrombosis at the ectatic coronary artery that dissolved after anticoagulation therapy prior to coronary angiography. Anticoagulant therapy may have a greater benefit than antiplatelet therapy in this patient due to the turbulence and stasis of blood in the ectatic vessel, although coexisting coronary conditions mandated antiplatelet therapy. His international normalized ratio was suboptimal and needed to be improved.

**Conclusion:**

Coronary ectasia may play a role in recurrent acute coronary syndrome, and administration of an anticoagulant to prevent acute coronary syndrome in this patient was in accordance with the varying hemodynamic property of coronary artery ectasia.

## Background

Coronary artery ectasia (CAE) is characterized by an abnormal dilatation of a coronary arterial segment of at least 1.5 times that of an adjacent normal coronary artery. CAE is observed in 3–8% of patients undergoing coronary angiography (CAG) and sometimes leads to acute coronary syndrome (ACS) regardless of the presence or absence of coronary stenosis or atrial fibrillation (AF) [1–3]. Approximately 20–30% of cases of CAE are thought to be congenital in origin (many of which coexist with coronary artery stenosis), 50% are attributable to atherosclerosis, and 10–20% are associated with inflammatory or connective tissue disease [1]. The slow flow phenomenon may lead to ischemia and thrombosis [4, 5]. These factors may then lead to ACS with varying pathophysiology that potentially requires a different approach.

The role of anticoagulants is still controversial in ACS with CAE. In this case report, we present a patient with recurrent ACS despite dual antiplatelet therapy (DAPT) and stenting of coronary arteries with significant stenosis in the presence of CAE. After intensified warfarin therapy, the patient was event-free at a 6-month follow-up, which indicated that anticoagulation is effective in such patients. To the best of our knowledge, at the time of this writing, our study was the first to report a fourth recurrence of ACS in a patient with right CAE.

## Case presentation

**Fig. 1:**
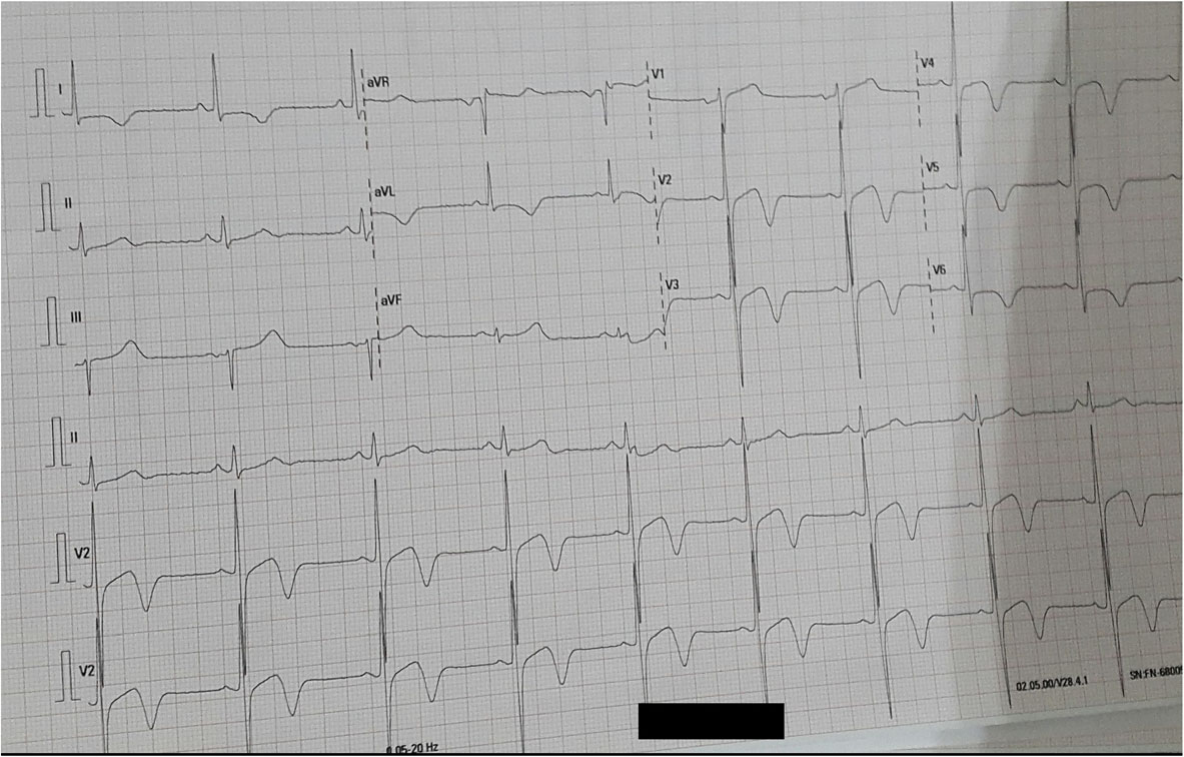
Electrocardiography at current admission. Sinus rhythm of 54 beats/minute, left ventricular hypertrophy, horizontal ST-segment depression, and T wave inversion at leads I, aVL, and V4–6, and a biphasic T wave at lead V2–3

A 61-year-old Indonesian man complained of typical chest pain that began 1 week before admission and had worsened 3 hours prior to admission. Accompanying symptoms were dyspnea, nausea, and sweating. On examination, his blood pressure was 110/80 mmHg, heart rate was 54 beats/minute, respiratory rate was 22 times/minute, and temperature was 37 °C. Cardiorespiratory examination results were within normal limits. A neurological examination was unremarkable. He had a history of dyslipidemia and hypertension, but there was no history of diabetes. His father had hypertension, but his family history was otherwise unremarkable. He quit smoking tobacco 17 months prior to admission. He did not drink alcohol. Current medications were captopril, bisoprolol, aspirin, clopidogrel, warfarin, isosorbide dinitrate (ISDN), and atorvastatin. He was not compliant with the warfarin regimen, particularly at a few weeks after hospital discharge and at 7 and 13 months before the present admission. Electrocardiography showed sinus rhythm of 54 beats/minute, left ventricular hypertrophy, horizontal ST-segment depression, and T wave inversion at leads I, aVL, and V4–6. A biphasic T wave was observed at lead V2–3 (Fig. 1). Laboratory results showed a suboptimal international normalized ratio (INR) of 1.28. The level of triglycerides was 273 mg/dL; low-density lipoprotein (LDL) and high-density lipoprotein (HDL) levels were within normal limits. The complete blood count and urea, creatinine, aspartate aminotransferase (AST), alanine aminotransferase (ALT), creatine kinase–myocardial band (CK-MB), and high-sensitivity troponin (hs-troponin) T levels were within normal limits. He was given a loading dose of aspirin and clopidogrel along with fondaparinux, and his symptoms were alleviated.

**Fig. 2:**
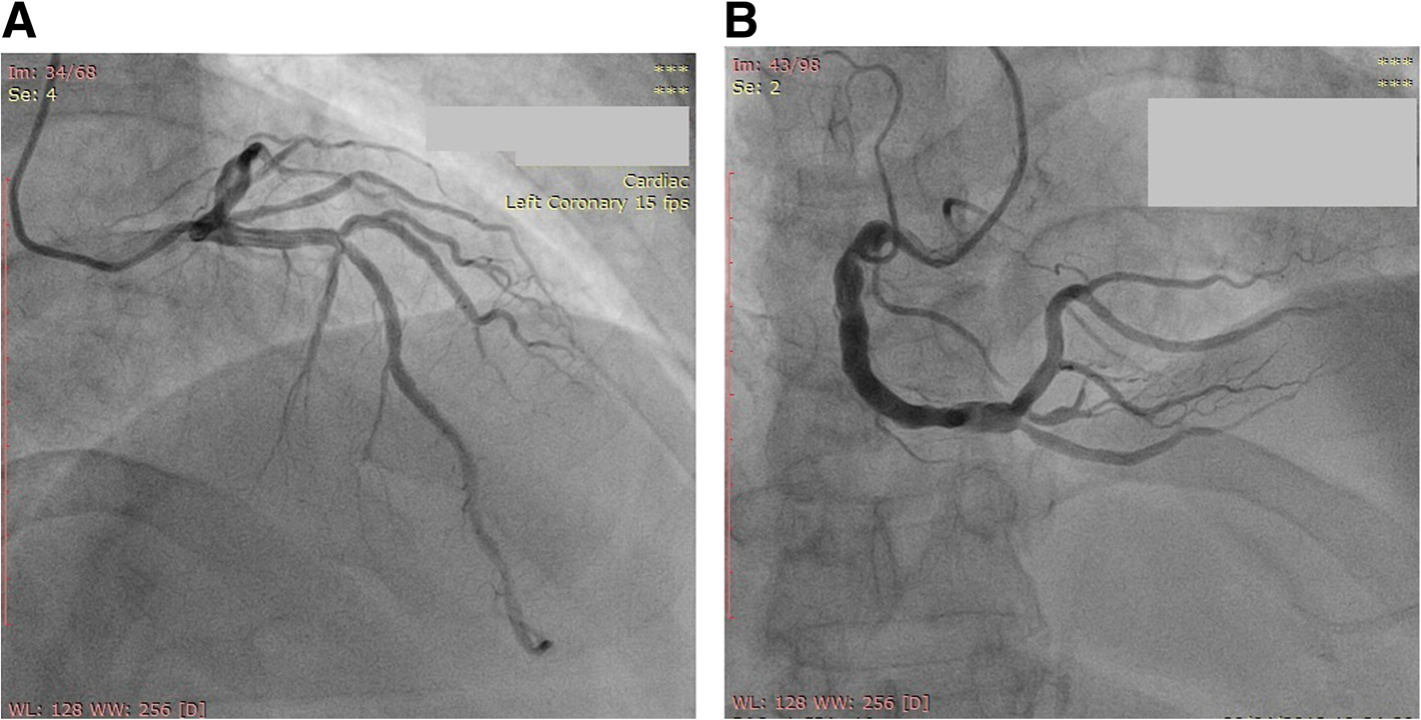
Angiography at current admission. Coronary angiography at the current presentation revealed two stents (**a**) and right coronary artery ectasia (**b**) without any significant obstruction

CAG showed an ectatic and turbulent mid-distal right coronary artery (RCA) and slow flow. There was a patent stent in the mid-left anterior descending coronary artery (LAD) and first diagonal branch (D1) (Fig. 2).

**Fig. 3:**
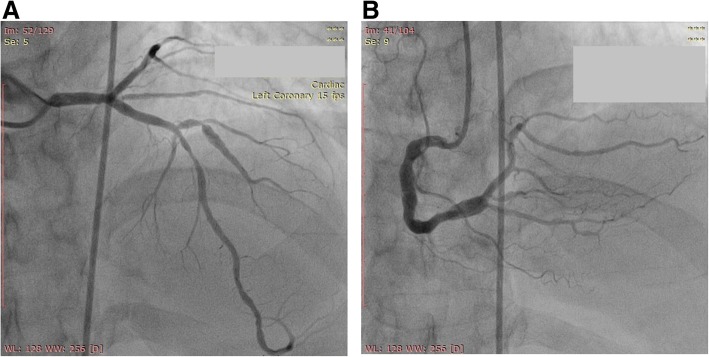
Angiography 17 months prior to present admission. Coronary angiography revealed 70% stenosis at the proximal D1 vessel and 50–60% stenosis mid-left anterior descending coronary artery past the D1 vessel (**a**) and ectatic, turbulent mid-distal flow and slow flow at the nonstenotic right coronary artery (**b**)

He had previously presented with chest pain on exertion (stable angina) at 17 months before the present admission. CAG showed an ectatic RCA, 30% irregular diffuse proximal-distal flow, turbulent distal flow, and 70% stenosis at the proximal D1 vessel and 50–60% stenosis at the mid-LAD past the D1 vessel (Table 1) (Fig. 3). He requested medical treatment rather than percutaneous coronary intervention (PCI); he was given bisoprolol, aspirin, ISDN, and atorvastatin and was then scheduled for a repeat angiogram 6 months later. However, he presented with unstable angina pectoris (UAP) 14 months before the latest presentation, ahead of the scheduled CAG. CAG showed a similar lesion at the RCA and 70% stenosis at the proximal LAD, 90% at the proximal D1 vessel. The D1 vessel was stented. He was given DAPT. He presented again with UAP 1 month later, and CAG showed a similar lesion at the RCA, 70–80% stenosis at the mid-LAD, and a patent D1 stent. PCI was performed, and the LAD was stented. His coagulation panel was within normal limits. He was given an anticoagulant due to angina caused by coronary ectasia. He presented again with UAP 7 months before the present admission, and CAG showed ectatic, turbulent mid-distal flow and slow flow in the dominant vessel, Thrombolysis In Myocardial Infarction (TIMI) flow II–III distal to the nonstenotic RCA, and a patent stent at the mid-LAD and D1 vessels. His INR was suboptimal (1.4). He was suspected of having recurrent ACS due to microvascular occlusion caused by slow flow and an ectatic vessel; warfarin therapy was intensified, and he was educated regarding the importance of reaching the INR target. At the time of the writing of this article, he had been event-free for 6 months, and his INR was 2.3. He remained compliant with the drug regimen.

**Table 1:** Summary of the patient’s chronology

Presentation	Features	Coronary Angiography	Antiplatelets/Anticoagulation
Current	UAP	Ectatic, turbulent mid-distal + slow flow without stenosis at RCA and patent stent at mid LAD and D1. INR was suboptimal (1.28)	DAPT + Warfarin
7 Months	UAP	Ectatic, turbulent mid-distal + slow flow without stenosis at RCA and patent stent at mid LAD and D1. INR was suboptimal (1.4)	DAPT + Warfarin
13 Months	UAP	Ectatic, turbulent mid-distal + slow flow without stenosis at RCA, a 70–80% stenosis at mid LAD, and a patent D1 stent. LAD stented	DAPT + Warfarin
14 Months	UAP	Ectatic, turbulent mid-distal + slow flow without stenosis at RCA, a 70% stenosis at proximal LAD, 90% at D1 prox. D1 was stented	DAPT
17 Months	SAP	Ectatic, turbulent mid-distal + slow flow without stenosis at RCA and 70% stenosis at D1 prox & 50–60% mid LAD after D1	Aspirin

*DAPT* Dual Antiplatelet Therapy, *INR* International Normalized Ratio, *LAD* Left Anterior Descending, *LCx* Left Circumflex Artery, *RCA* Right Coronary Artery, *SAP* Stable Angina Pectoris, *UAP* Unstable Angina Pectoris.

## Discussion

This patient had right CAE with stents in significant coronary lesions. He experienced recurrent ACS while on DAPT and suboptimal warfarin therapy despite patent stents and the absence of an obstructive coronary lesion. To the best of our knowledge, at the time of this writing, the present case was the first to report a fourth recurrence of ACS in a patient with right CAE despite the absence of obstructive lesions upon angiography. After intensified warfarin therapy, which resulted in an INR of 2.3, our patient was event-free for 6 months, highlighting the importance of anticoagulant therapy in a patient with recurrent ACS and CAE without obstructive lesions.

Initially, the angina was thought to be solely caused by obstructive coronary artery disease due to the presence of significant stenosis in the coronary vessel. After stenting the vessel, our patient presented again with ACS, and the second stent was deployed. He received DAPT along with warfarin after the second stenting. However, he presented with UAP at 7 months before latest admission and at the latest admission despite a patent stent and no other significant obstructive lesion. This observation led to the suspicion that both the CAE and coronary stenosis played a role in the recurrent acute pain. ACS in patients with CAE has varying pathophysiology and may be divided into pathology that is detectable by CAG, such as visible thrombosis of the epicardial coronary artery, or not detectable by CAG, such as slow flow, vasospasm, and thrombosis in the microvasculature. In our patient, there was an absence of a flow-limiting lesion, indicating that the ACS may be due to the latter type of CAE, especially the coronary slow flow (CSF) phenomenon [6]. Delayed distal vessel opacification in the absence of significant epicardial coronary artery disease is characteristic of CSF. CSF may cause myocardial ischemia and injury, especially during stress, which is thought to be related to coronary endothelial dysfunction [7, 8]. CSF might also be caused by microvascular angina due to small thrombi that are not visible or microvascular spasms during CAG. CSF might also be caused by a thrombus in the ectatic vessel that dissolves prior to CAG due to anticoagulant treatment and a loading dose of DAPT, as the symptoms were alleviated after drug administration.

The goal of therapy is to prevent further myocardial ischemia and minimize the risk of thrombosis due to an inflammatory state and slow flow. Thrombosis at the ectatic coronary artery is more likely to be a high burden and result in poor reflow after intervention, with poor outcomes [9]. Treatment with warfarin itself has not been prospectively studied, and the recommendation is not clear. In patients with isolated CAE, anticoagulant therapy alone may be appropriate due to turbulence and stasis of blood in the ectatic vessel [6]. A search of existing literature that reported cases of patients with recurrent ACS in CAE showed that there were two isolated cases of CAE in which the patient took DAPT without an anticoagulant and experienced recurrence [10–18]. To the best of our knowledge, there has not been a report of recurrent ACS after anticoagulation that has met the target of treatment, and there has not been a report of recurrent ACS after treatment with DAPT in a nonisolated case. Our patient did not have any significant lesion at the current presentation, and the pain may be due to “isolated CAE”; hence, anticoagulation might be helpful in our case. Interestingly, after being given warfarin, our patient was event-free for 6 and 7 months, whereas previously, the recurrence occurred at a shorter timeframe, coinciding with him becoming noncompliant a few weeks after warfarin was given (Table 1). However, he also had coexisting coronary artery disease and a stent in the coronary artery requiring DAPT. Because the benefit outweighed the risk of bleeding in this patient, triple therapy with aspirin, clopidogrel, and warfarin was continued. The INR in this patient was suboptimal and needed to be improved. Nitrates may exacerbate stress-induced myocardial ischemia by causing epicardial vasodilation in isolated CAE. Nitrates were to be used conservatively in this patient because the obstructive lesion had been addressed, and it was possible that the angina was solely due to CAE, which may be exacerbated by the administration of nitrates. Calcium channel blockers and beta-blockers should thus be the mainstay anti-ischemic vasodilator therapy in our patient [6].

## Conclusion

CAE may cause recurrent ACS with an obstructive or nonobstructive lesion on CAG. Administration of an anticoagulant should be considered to prevent ACS in these patients without ignoring the need for antiplatelet therapy in the presence of atheroma. Nitrates should be avoided in cases of pure CAE and used sparingly in those with coexisting coronary artery disease. Beta-blockers and calcium channel blockers are preferred over nitrates as anti-ischemic vasodilator agents.

### Patient’s perspective

The patient admitted that it is difficult to comply with the drug regimen because he has to take several pills a day. He also feared that taking too many pills would lead to renal damage. After reemphasizing the importance of anticoagulant therapy, the patient understood and planned to enlist the help of family members to remind him to take his medication and help him keep a diary.
